# T Lymphocyte–Endothelial Interactions: Emerging Understanding of Trafficking and Antigen-Specific Immunity

**DOI:** 10.3389/fimmu.2015.00603

**Published:** 2015-11-24

**Authors:** Christopher V. Carman, Roberta Martinelli

**Affiliations:** ^1^Center for Vascular Biology Research, Department of Medicine and Emergency Medicine, Beth Israel Deaconess Medical Center, Harvard Medical School, Boston, MA, USA

**Keywords:** lymphocyte, TCR, Trafficking, endothelium, antigen presentation, MHC, immunomodulation, tolerance

## Abstract

Antigen-specific immunity requires regulated trafficking of T cells in and out of diverse tissues in order to orchestrate lymphocyte development, immune surveillance, responses, and memory. The endothelium serves as a unique barrier, as well as a sentinel, between the blood and the tissues, and as such it plays an essential locally tuned role in regulating T cell migration and information exchange. While it is well established that chemoattractants and adhesion molecules are major determinants of T cell trafficking, emerging studies have now enumerated a large number of molecular players as well as a range of discrete cellular remodeling activities (e.g., transmigratory cups and invadosome-like protrusions) that participate in directed migration and pathfinding by T cells. In addition to providing trafficking cues, intimate cell–cell interaction between lymphocytes and endothelial cells provide instruction to T cells that influence their activation and differentiation states. Perhaps the most intriguing and underappreciated of these “sentinel” roles is the ability of the endothelium to act as a non-hematopoietic “semiprofessional” antigen-presenting cell. Close contacts between circulating T cells and antigen-presenting endothelium may play unique non-redundant roles in shaping adaptive immune responses within the periphery. A better understanding of the mechanisms directing T cell trafficking and the antigen-presenting role of the endothelium may not only increase our knowledge of the adaptive immune response but also empower the utility of emerging immunomodulatory therapeutics.

## Introduction

To fulfill their roles of conducting immune surveillance and mediating immune responses, cells of the immune system (leukocytes) must continuously traffic into and out of the tissues and vascular–lymphatic circulation. In the case of T lymphocytes (T cells), this trafficking is required to perform repeated serial encounters with diverse host cells in search of cognate peptide antigens presented on their surface (1). The endothelial cell lining of the contiguous vascular–lymphatic circulatory system is a highly unique anatomical structure; it serves both as a physical barrier that separates the tissue and blood–lymph compartments as well as as an interface for communication between the two. Thus, leukocyte–endothelial interactions are of special importance for the regulation of the immune system. Albeit with high degree of local diversity and heterogeneity, collectively these serve as critical rate-limiting determinants of immune cell trafficking across the endothelial barrier (a process known as “transendothelial migration” or “diapedesis”) (1, 2). Moreover, close interactions between immune cells and the endothelium are increasingly appreciated to influence the quality of immune responses through diverse modes of information exchange including antigen presentation.

In this review, we provide an overview of the basic features of the vascular–lymphatic circulation and T lymphocyte trafficking, summarizing the expanding knowledge of the subcellular dynamics that regulate diapedesis. After establishing this background, we discuss the known functional contributions of endothelial cells to the regulation of adaptive immunity, the unique cellular and molecular mechanisms involved in this process, and their potential relevance to immunopathologies and emerging immunomodulatory therapy.

## Endothelia and Vascular–Lymphatic Circulation

**Figure 1:**
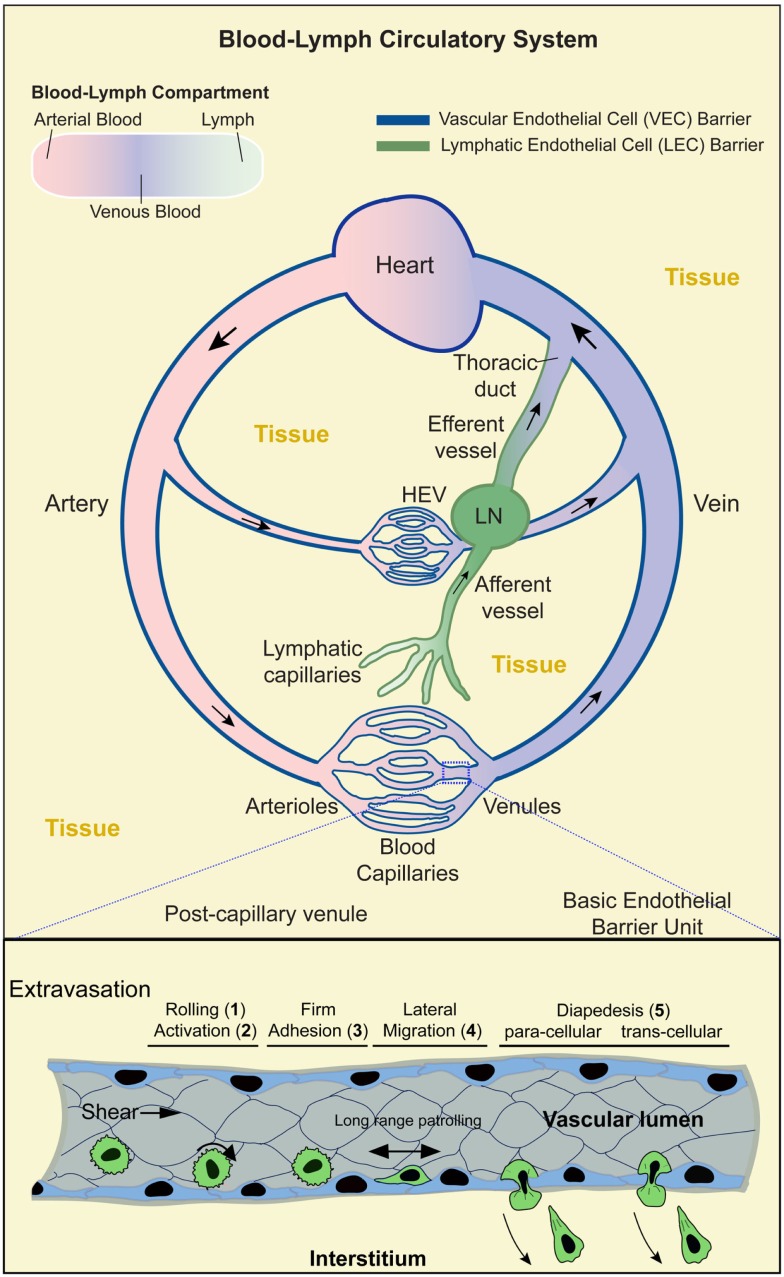
**Blood–lymph circulatory system and lymphocyte trafficking**. Upper panel: schematic shows the contiguous blood–lymph circulatory system. Arterial, oxygen-rich, blood (pink) flows away from the heart and into the microvasculature (arterioles, capillaries, and venules). Oxygen-depleted blood (blue) flows from the microvasculature back to the heart. Lymph (green) collected from the tissues (yellow) is taken up by the lymphatic capillaries to flow through the afferent lymphatic vessels, lymph nodes (LN), efferent vessels, and back into the blood circulation via the lymphatic duct. Local microvasculature of the LN (i.e., high endothelial venules; HEV) serves as a location for lymphocytes to enter the LN. Dark blue and dark green lines indicate the vascular and lymphatic endothelial barriers, respectively. Boxed region (lower panel) shows a segment of a postcapillary venule during the process of lymphocyte extravasation. This process evolves in stages: (1) transient rolling interactions mediated predominantly by selectins; (2) subsequent chemokine-dependent activation; (3) firm arrest, which is mediated by the binding of lymphocyte integrins (e.g., LFA-1, Mac-1, and VLA-4) to endothelial cell-adhesion molecules (e.g., ICAM-1, ICAM-2, and VCAM-1); (4) lymphocyte lateral migration on the surface of the endothelium, probing for a site to penetrate through it (tenertaxis); and (5) Lymphocytes diapedesis across the endothelial barrier to enter the interstitium either following the paracellular route (by opening a gap between two adjacent endothelial cells) or transcellular route (by migrating directly through the body of a single endothelial cell).

The body is functionally organized through a multitude of compartmentalization schemes that are diverse in scale and architecture. In vertebrates, which possess a closed cardiovascular system, the two most rudimentary compartments are the tissues and the contiguous vascular (or blood)–lymphatic circulation (3) (Figure 1). The latter is essentially a series of liquid (and blood cell) filled vessels that are densely interdigitated throughout the tissues. The endothelium is the cellular lining of these vessels, providing the essential barrier that establishes and maintains the separate tissue and blood–lymph compartments (Figure 1, lower panel). In all cases, the minimal barrier unit consists fundamentally of a monolayer of vascular (VEC) or lymphatic (LEC) endothelial cells that are bound to each other by adherens and tight junction proteins (e.g., VE-cadherin, PECAM-1, JAM-1, CD99, claudins, and occludins) and to the underlying matrix by integrin adhesion receptors (e.g., αvβ3 and α5β1) (4–6) (Figure 1).

Overall, the vascular component of the circulatory system is comprised of a series of large (macrovascular) arterial vessels that carry oxygen-rich blood away from the heart and progressively branch into smaller diameter arteriole vessels and finally capillaries where oxygen and nutrient are exchanged (Figure 1). Downstream of the capillary beds, individual vessels progressively merge into larger postcapillary venules and veins that eventually return to the heart. In total, the vascular endothelium represents an enormous surface area [~4000–7000 m^2^ (7, 8)], whereby all vascularized tissues are densely packed with microvessels such that most tissue cells lie within tens of microns of a vessel (9, 10). An important consequence of this arrangement is that every tissue microenvironment has a local interface (i.e., VECs) with the circulation.

The lymphatic component of the circulatory system runs parallel to, works with, and is physically contiguous with the cardiovascular component. Here, however, instead of forming a closed circulation, the lymphatic vessels begin blindly in the tissue (akin to tree roots; Figure 1) (11). The initial vessels (terminal lymphatic capillaries) merge progressively to form afferent collecting vessels that feed into secondary lymphoid organs (SLOs; LN, spleen, Peyer’s patch, and tonsils). Downstream of the SLO is efferent lymphatic vessels that merge into collecting vessels and finally into the thoracic duct that connects to the venous blood circulation via the subclavian vein (Figure 1). The lymphatic system maintains fluid homeostasis by providing a conduit through which tissue fluids, derived from inherently leaky blood capillaries, are collected and returned to the vascular circulation. This also serves as a route by which tissue antigens are either passively (in the lymph fluid) or actively [via trafficking of phagocytic interstitial antigen-presenting cells (APCs), e.g., dendritic cells; DCs] delivered to SLOs (11) (Figure 1).

In addition to the essential roles in tissue oxygenation and fluid homeostasis, vascular–lymphatic circulation and endothelia should be viewed as critical non-hematopoietic components of the immune system. The basic immune surveillance and response function of leukocytes demands continuous and efficient trafficking throughout the body (1). The vascular–lymphatic circulation provides a means of rapid and organized transit of immune cells. As discussed below, immune cells must also repeatedly leave (extravasate) and re-enter (intravasate) the circulation in diverse settings, which requires them to continuously migrate through endothelial barriers (a process known as “transendothelial migration” or “diapedesis”) (1, 2). The immune cells and the endothelium have coevolved complex biochemical and biomechanical information exchange systems that drive selective diapedesis of leukocytes with high efficiency and precise spatiotemporal control. Emerging understanding of endothelial as “semiprofessional” APCs further suggests non-redundant roles in shaping adaptive immune responses.

## An Overview T Lymphocyte Function and Trafficking

T cells are the part of the adaptive immune system that surveys the tissues for the presence of infecting pathogens (e.g., virus or bacteria) or aberrant host cells (e.g., tumor cells), specifically by detecting non-self/abnormal protein fragments (i.e., peptide antigens; Ag) through specialized cell surface T cell receptors (TCRs). Critically, peptide Ag can only be sensed when presented on the surface of another cell by major histocompatibility complex (MHC) proteins (12, 13). T cells that express the cell surface marker CD4 only respond to Ag presented on class II MHC molecules (MHC-II) and participate in immune reactions mostly through secretion of cytokines that “help” other immune cells to conduct their functions (termed “CD4 T helper or Th cells”) (12, 13). Those that bear CD8 respond to Ag presented on MHC-I and function in direct killing of Ag bearing cells (termed “CD8 T cells” or “cytotoxic T cells”; CTLs) (12, 13). T cells that have never encountered their specific cognate Ag are termed “naive. ” Those that have been activated by Ag become either “effector cells” that participate actively in immune responses to eradicate the detected pathogen or long-lived “memory” cells that serve as the basis for “acquired” or “adaptive” immunity.

The process of T cell maturation, surveillance, and responses requires diverse trafficking patterns and interactions with the endothelium. T lymphocytes originate from stem cells in the bone marrow that become lymphoid precursors. These first intravasate across the bone marrow endothelium (14–16) and thereby enter the blood circulation to immediately home and extravasate into the thymus and complete their maturation. Ultimately, mature naive, non-self-reactive lymphocytes re-enter the blood and initiate immune surveillance by engaging in constitutive cycles of migrating into and out of SLOs in search of cognate Ag. Inside the LN, T cells patrol the “T cell area” where they form serial short-lived intimate contacts with the DCs that allow them to survey the cell surface MHC–Ag complexes (17, 18). In the absence of cognate Ag recognition, T cells exit the LN, mediating extensive interactions with the LEV that line the cortical and medullary sinuses of the LN, as well as the efferent lymphatic vessels that carries them back into the blood circulation (19–22). When cognate MHC/Ag is encountered [in concert with the appropriate combination of self-surface costimulatory and coinhibitory molecules and cytokines (12, 13, 23–25)] by T cells, they engage in extended (~30–60 min) intimate cell–cell interactions with DCs, which triggers their activation (e.g., calcium flux and nuclear translocation of the transcription factor NFAT), proliferation, and differentiation into Ag-specific effector and memory lymphocytes (26–29). These cells re-enter the circulation reprogramed (e.g., with decreased CD62L and CCR7 and increased LFA-1, VLA-4, CCR3, CCR5, and CXCR3 expressions) to adhere preferentially to, and diapedeses across, activated endothelia of infected/inflamed peripheral tissues (1, 30).

In addition to the above processes, emerging intravital imaging technologies have begun to reveal settings in which sustained intra- and extravascular T cell–endothelial interactions take place that are not strictly associated with diapedesis. For example, CD4 Th1 and Th17 lymphocytes were discovered to undergo repeated transient (~20 min duration) arrest on resting liver sinusoidal endothelium (31). Additionally, natural killer T cells (32, 33) and effector T lymphocytes were shown to undergo extended both luminal and abluminal long-range “patrolling migration” on liver sinusoidal and brain microvascular endothelium, respectively (34). Taken together, the above discussion highlights a multitude of diverse T cell–endothelial interactions that allow for information exchange that can influence the trafficking, activation, and differentiation of lymphocytes.

### Heterogeneity in Lymphocytes, Endothelium, and Their Interactions

As noted above, there are many different scenarios for lymphocyte/endothelial interactions. It is critical to appreciate that enormous contextual heterogeneity exists spatially and temporarily in such encounters. Of course it is well established that lymphocytes take on diverse characteristics as naive, effector, and memory phenotypes evolve. Moreover, a large (and still expanding) collection of differing subsets exists [e.g., CD4 effectors include Th1, Th2, Th9, Th17, Th22, Tregs, Tfh, and Tfr (35)]. Collectively, these exhibit specialization in their protein expression that give rise to diverse trafficking patterns and responses to their local environment. Perhaps the most obvious example, naive lymphocytes avidly and perpetually enter LN via their specialized high endothelial venules (HEV) but ignore activated microvasculature of other tissues. Moreover, effector memory lymphocytes show great tissue selectivity driven in particular by surface expression of specific chemokines and/or adhesion molecules both on their surface and ECs. For example, gut-specific lymphocytes selectively express the integrin α4β7 and the chemokine receptor CCR9, which binds the adhesive ligand MadCAM and the chemokine CCL25, which are selectively presented on HEV and microvasculature of the intestine (36, 37). While we will expand on some of these topics, these differences have been extensively documented and comprehensively reviewed elsewhere (1, 35, 38, 39).

The endothelium exists to subserve the tissues it perfuses. As such it takes on specialized form and function to both adapt to its microenvironment and meet the local and diverse needs. Such heterogeneity can be observed at multiple levels in structure and in function. Major differences exist in arterial versus venous aspects of the circulation, where, for example, large differences in oxygen tension and pH exist (7). Additionally, macrovascular structures that serve dominant conduit functions experience far greater fluid shear forces compared to the microvasculature that plays a major role in the regulation of selective permeability and the control of immune surveillance. Furthermore, vascular beds of different tissues exhibit widely different features (9, 10). For example, microarray analysis comparing endothelia of either macro- versus microvasculature or arterial versus venus circulation and of different tissues has demonstrated distinction in cell surface adhesion molecules, cell surface signaling receptors, intracellular signaling molecules, and cytoskeletal proteins (40). Thus, different endothelial cells will differ in their morphology and their ability to sense and respond to various stimuli. In particular, with respect to immune system function, it is important to note that in most tissues (with the exception of lung and liver, see below), the postcapillary venules are distinctly responsive to inflammatory cytokines and therefore selectively increase expression of adhesion molecules that bind leukocytes (e.g., E-selectin, P-selectin, ICAM-1, and VCAM-1). As such these function as a major site for leukocyte–endothelial interactions and leukocyte transendothelial migration. Distinct lymphocyte/endothelial interactions are also seen along the microvasculature of different tissues. For example, HEV of lymphoid organs selectively expresses adhesion molecules (e.g., peripheral node addressin; ligand for L-selectin) and chemokines (e.g., CCL21; ligand for CCR7) to promote homing of naive lymphocytes, whereas intestinal microvascular express molecules to selectively recruit gut-specific effectors as noted above. Additionally, the tight junctions of the brain microvasculature significantly limit leukocyte trafficking and thereby contribute to the immune privilege of the CNS. On the other hand, the relatively porous (fenestrated and sinusoidal) endothelium of the liver allows luminal lymphocytes to continually probe underlying APCs (e.g., Kupffer cells) to receive tolerogenic signals from food antigens.

Collectively, the above points to the critical idea that all lymphocyte and endothelial interactions are not created equally. Said another way, the phenotype of any given lymphocyte and a given endothelial cell represents the product of each of their microenvironments/experience, which drives potentially vastly different outcomes of specific lymphocyte/endothelial interactions. Relevant microvascular cues are diverse and numerous including, for example, cytokines, pathogens, tissues damage, oxygen tension/hypoxia, and biomechanical strains. Such selective outcomes should be viewed as the critical underpinning of a well-orchestrated immune response and therefore of exceeding importance. That being said, this review cannot possibly hope to catalog all of the potential combinations and their outcomes. Equally important, the vast majority of these combinations have yet to be systematically explored. Thus, in the following sections, we will focus on well-established themes and attempt to highlight illustrative examples of heterogeneity where possible.

## The Multistep Cascade of Lymphocyte Diapedesis

Lymphocyte trafficking across the endothelium is a tightly controlled process that requires highly orchestrated dynamics on the part of both the lymphocyte and the endothelium. Though the process of intravasation and extravasation is equally important and probably share many the mechanisms (16, 41, 42), only the latter has been characterized in detail as discussed herein. The initiating events in extravastion (whether for constitutive or inflammation-induced trafficking) are the active cytokine-driven expression and cell surface presentation of chemoattractants and adhesion molecules by endothelial cells (1, 2, 43–47). This sets the stage for a well-ordered multistep cascade of lymphocyte adhesion and diapedesis (Figure 1, lower panel).

Lymphocytes entering a postcapillary venule of a LN or an inflamed tissue first undergo transient tethering and rolling interactions with the endothelium mediated by the selectin family of adhesion molecules binding to their glycoprotein/glycolipid ligands (step 1; Figure 1, lower panel) (30, 48, 49). This promotes increased cell–cell contacts that facilitates lymphocyte sensing of chemokines presented on the endothelial surface, which in turn induces intracellular signaling responses (step 2) leading to high affinity interaction of lymphocyte integrin adhesion receptors (e.g., LFA-1, Mac-1, and VLA-4) with their endothelial ligands (e.g., ICAM-1, ICAM-2, and VCAM-1) and subsequent firm lymphocyte arrest (step 3) (50). Lymphocytes then undergo actin-dependent spreading, polarization, and integrin/CAM-dependent lateral migration over the luminal surface of the endothelium (step 4). This apparently allows T cells to search for sites permissive for endothelial barrier breach (51, 52). Ultimately, lymphocytes penetrate the endothelium either at the intercellular junctions (i.e., paracellular migration) or directly through individual endothelial cells (i.e., transcellular migration) and move into the tissues (step 5) in a process mediated by integrins, CAM ligands, and other adhesion molecules, such as PECAM-1, JAM-1, and CD99 (2, 30, 48, 49, 53, 54).

Although the above cascade is widely applicable, alternatives and exceptions exist. For example, in lung and liver (two particular large and important vascular beds), extravasation is observed to occur at the capillaries, typically through a rolling-independent process (9, 10). Moreover, as noted above, subsets of lymphocytes have been observed to undergo extensive patrolling migration on endothelium (step 4) without necessarily progressing to transmigration (32–34).

## T Cell Remodeling During Adhesion and Diapedesis

Function blocking antibodies and other pharmacologic and genetic approaches have been instrumental for the elucidation of the above cascade and the identification of an ever-growing list of chemoattractant, adhesion, and signaling molecules that participate in trafficking (55). More recently, high-resolution live-cell fluorescence imaging has begun to reveal new subcellular dynamic behaviors that underlie the orchestrated process of lymphocyte diapedesis. As noted above, initial arrest of T cells on endothelium is followed by dramatic cytoskeletal rearrangements leading to rapid spreading and polarization, with the prominent appearance of a trailing uropod and a leading edge lamellipodia that promote lateral migration over the endothelium.

**Figure 2:**
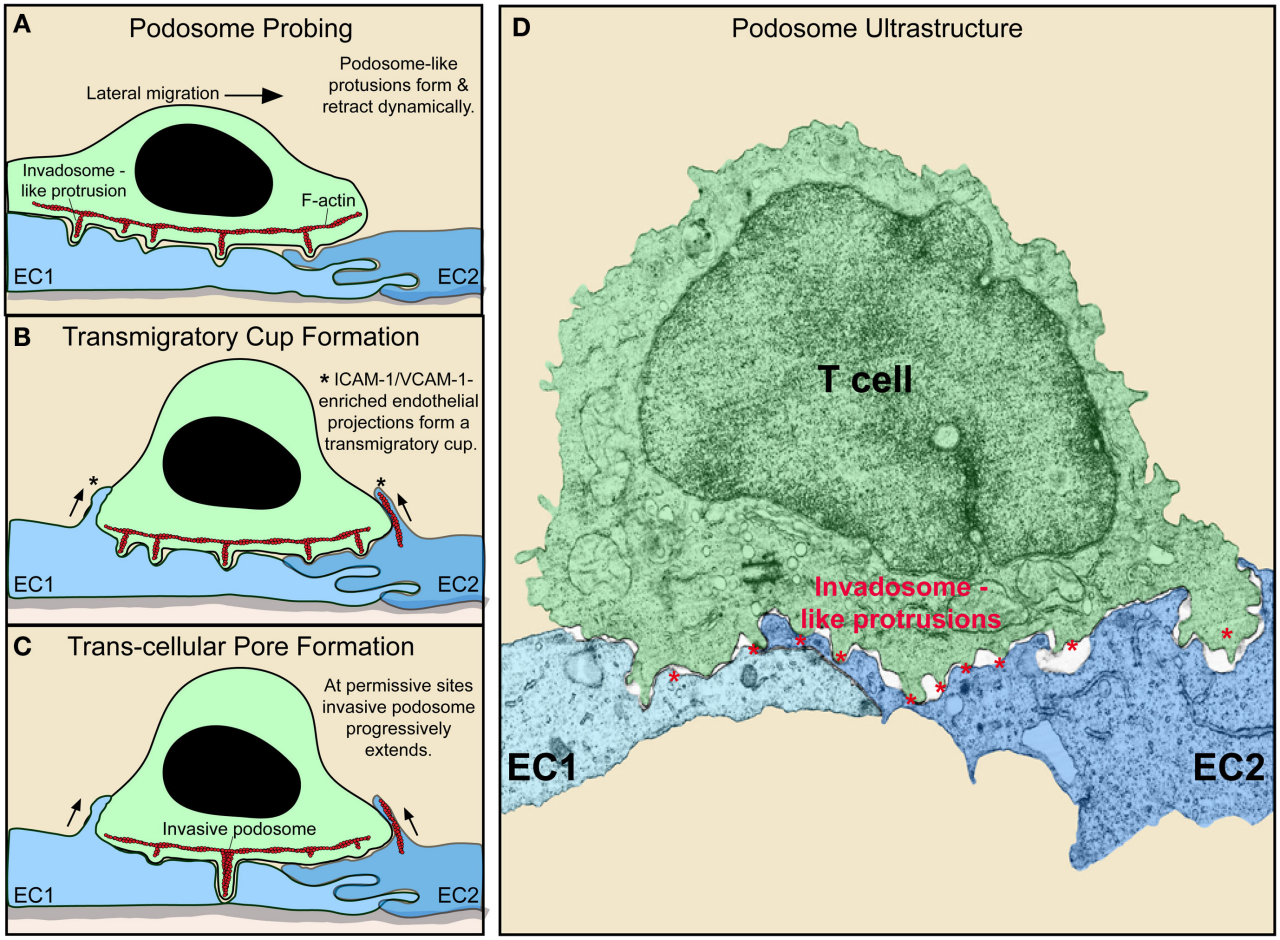
**Dynamic remodeling of lymphocytes and endothelium during diapedesis (A–C)**. Schematic shows lymphocyte (green) and EC (blue) dynamics during T cell lateral migration over, and transcellular diapedesis across, the endothelium. **(A–C)** Successive time points at intervals of ~30–60 s. Dynamic insertion (~0.2–1 μm in depth) and retraction of multiple actin-rich lymphocyte invadosome-like protrusions (ILPs) into the apical surface of the endothelium occurs during lateral migration **(A–C)**. Once a location of sufficiently low endothelial resistance has been identified (tenertaxis), an ILP progressively extends several micrometers in depth, ultimately breaching the endothelium transcellularly **(C)**. Also shown is the “transmigratory cup” structure (asterisks), which consists of vertical endothelial microvilli-like projections (rich in F-actin; red, ICAM-1, VCAM-1, PECAM-1, and JAM-1) that surround the periphery of adherent lymphocytes **(B–D)**. Electron micrograph of a T cell (green) extending multiple ILP (red asterisks) into the surface of two endothelial cells (EC1, EC2; blue) near an intact junction in order to probe for a site to initiate diapedesis (i.e., breach the endothelial barrier) (60).

It has become recognized that during spreading and lateral migration, lymphocytes generate discrete actin-rich micron-scale projections that extend in the direction orthogonal to the plane of migration (42, 56–58) (Figure 2). These cylindrically shaped structures, termed invadosome-like protrusions [ILPs; related to podosomes and invadopodia found in other cells types (59)], protrude from the bottom of the T cells, exerting mechanical forces against the surface of the endothelium. This drives extremely close cell–cell contacts that lead to sharp indentations in the endothelium (termed “podo-prints”) (56) (Figure 2). ILPs are enriched in, and functionally require, LFA-1, the actin regulatory proteins WASp and HS1 (a hematopoietic homolog of cortactin) and src kinase (42). As T cells migrate, they continuously extend and retract clusters of ILPs against the endothelium that rapidly turnover (life-times of ~20 s). In this way, lymphocytes effectively probe or “palpate” the local biomechanical properties (e.g., stiffness) of the endothelial substrate as they move over it (Figure 2).

Such “biomechanical scanning” facilitates the stochastic identification of regions of endothelial surface that are sufficiently tenuous to allow ILPs to progressively extend and formally breach the endothelial barrier and thereby initiate diapedesis (56, 60). Thus, lymphocytes seem to employ ILPs in an active process of seeking out the path-of-least-physical-resistance for egress into (or out of) the tissue (termed “tenertaxis”; from the latin tener, soft) (60). Lymphocytes deficient in WASp [i.e., as is found in patients with the genetic immune-deficiency known as Wiskott–Aldrich syndrome (WAS)] spread and laterally migrate normally but fail to form ILPs, which effectively results in defective tenertaxis that in turn leads to inefficient diapedesis (56).

Additionally, investigations have shown that the extremely intimate cell–cell contacts (which are normally opposed by formidable electrostatic and steric repulsion forces inherent in the cell glycocalyx) that are driven by ILPs allow lymphocytes to detect discrete pools of chemokine that are held close to the endothelial plasma membrane (61). Thus, lymphocyte ILPs may function both for biomechanical and biochemical probing of the endothelial surface (as discussed further below). Of note, lymphocyte ILPs have been widely evidenced ultrastructurally *in vivo* in virtually all lymphocyte–endothelial interaction settings (e.g., bone marrow, thymus, HEVs, SLOs, and diverse inflamed tissues) including both intravasation and extravasation events (14, 16, 21, 22, 54, 62–72). Thus, ILPs may represent a broadly relevant sensory organelle that lymphocytes use to continuously probe their local cellular environment as they traffic.

## Endothelial Remodeling During T Cell Adhesion and Diapedesis

While the endothelium was once considered an inert membrane, it is now clear that it actively responds to lymphocyte adhesion and is involved in guidance during the process of diapedesis. As noted above, the endothelium plays active, carefully controlled roles in the expression and presentation of chemoattractans and adhesion molecules. Additionally, detailed imaging studies have revealed avid local cytoskeletal remodeling at the site of contact with T cell. Specifically, upon adhesion of lymphocytes (or other leukocyte types), integrin-mediated (i.e., LFA-1, Mac-1, and VLA-4) binding and resultant clustering of endothelial ICAM-1 and VCAM-1 induce rapid formation of actin-dependent microvilli-like protrusions via signaling through the ERM family of cytoskeletal adaptor proteins (73–76) (Figures 2A–C). During rapid lateral T cell migration, these microvilli contacts tend to form asymmetrically, serving as tethers attached at the uropod of the lymphocyte encouraging lateral migration arrest (75) (Figure 2A). As the lymphocyte slows its lateral migration, endothelial microvilli form more symmetrically around it to effectively embrace it forming a cuplike structure known as “transmigratory cup” (Figures 2B,C). This results in an expansion of cell–cell contact area that is coenriched in LFA-1, VLA-4, ICAM-1, and VCAM-1. Such an arrangement strengthens adhesion to resist fluid shear forces and also provides an adhesion scaffold oriented perpendicular to the plane of the endothelium that guides and facilitates diapedesis (76). Another important consequence of (or role for) the transmigratory cup structure is that the resultant extended close cell–cell contacts should promote maintenance of effective endothelial barrier (i.e., with respect to fluid and solutes) during diapedesis. In this regard, recent studies have elucidated a further active endothelial remodeling process that assures rapid resealing of the endothelium at the conclusion of a given diapedesis event. Specifically, it was demonstrated that the endothelium restores its integrity by mobilizing an integrin-, Rac-1-, and Arp2/3-dependent actin-rich “ventral lamellipodia” that rapidly re-seals the endothelial barrier from its ventral surface (77). Thus, endothelial cells actively support and guide lymphocyte egress across itself while maintaining barrier integrity through intimate adhesions and actin remodeling dynamics.

## Endothelium as a Regulator of Immune Cell Activation and Differentiation

As discussed above, clearly the endothelium is a critical regulator of immune cell trafficking. However, it is also clear that the endothelium acts as a sentinel (e.g., to relay local tissue status signals) in ways that additionally influence immune cell activation and differentiation states. Studies in a range of innate and adaptive immune cells have established that diapedesis across inflamed endothelium has broadly proinflammatory or “priming” effect on these cells (78). On the contrary, other studies (discussed in part below) suggest settings whereby endothelial encounter may impart anti-inflammatory or tolerogenic effects. Such reports suggest that the endothelium, which is strategically positioned as the blood–tissue interface and an obligate interaction partner for trafficking immune cells, can serve as critical checkpoint for adjusting or controlling immune reactions. Importantly, as integrators of their local environment, endothelia exhibit local heterogeneity that should be expected to contribute to the specific outcomes of such interactions. In the following section, we will review the emerging evidence for unique, non-redundant roles of endothelia as peripheral/stromal, semiprofessional, non-hematopoietic, APC (nhAPC).

## A Functionally Distinct Peripheral Non-Hematopoietic APC Compartment

Hematopoietic APCs, DCs in particular, play absolutely essential roles in the initiation and shaping of adaptive immune responses. However, the use of bone marrow chimeras (among other approaches) has led to the discovery of a functionally important peripheral, “non-hematopoietic” (“parenchymal,” “stromal”) compartment of APCs (nhAPCs). These play critical and distinct roles that complement those of hematopoietic APCs (79–84). Indeed, using bone marrow chimeras in combination with whole animal genetic knock-out strains (e.g., of the APC coinhibitor molecule PD-L1), essential contributions of nhAPCs have been revealed in promoting tolerance in settings of diabetes (83, 85), atherosclerosis (86), organ transplant (87, 88), myocarditis (89), and EAE (90). Similar studies have established unique roles for nhAPCs in mitigating tissue damage during systemic viral infection (91, 92), suppression of lung inflammation (93), and driving graft-versus-host disease (94). Multiple cell types have been identified as putative members of this “stromal” APC compartment including endothelial cells, fibroblasts, myofibroblasts, pericytes, smooth muscle cells, and mesenchymal stem cells (79–81). Among them, the strongest evidence suggests the endothelium as at least one of the key cell types that contribute to the identified nhAPC function.

## The Endothelium as a Unique nhAPC

Induction of a T cell response requires three canonical signals to be provided by the APC: (1) cell surface cognate peptide Ag in complex with MHC-I and -II (implicit in the activity is the ability to both phagocytose and process Ag); (2) cell surface costimulatory ligands; and (3) secreted cytokines. The appropriate combination of all three of these inputs is required to activate naive T cells (12, 13, 23–25). The quality of each of these signals determines the strength and type of responses generated (e.g., proinflammatory Th1 versus anti-inflammatory/tolerogenic CD4 regulatory T cells; Treg). Once primed and expanded, the resultant effector T cells re-enter the circulation to home to peripheral sites of inflammation in search of cognate Ag on interstitial APCs and targets. Thus, an obligate step in this process is the adhesion of the effector T cell to the endothelium. As described below, endothelial are equipped with all of the necessary capabilities (with one key exception) to provide APC signals 1–3.

**Figure 3:**
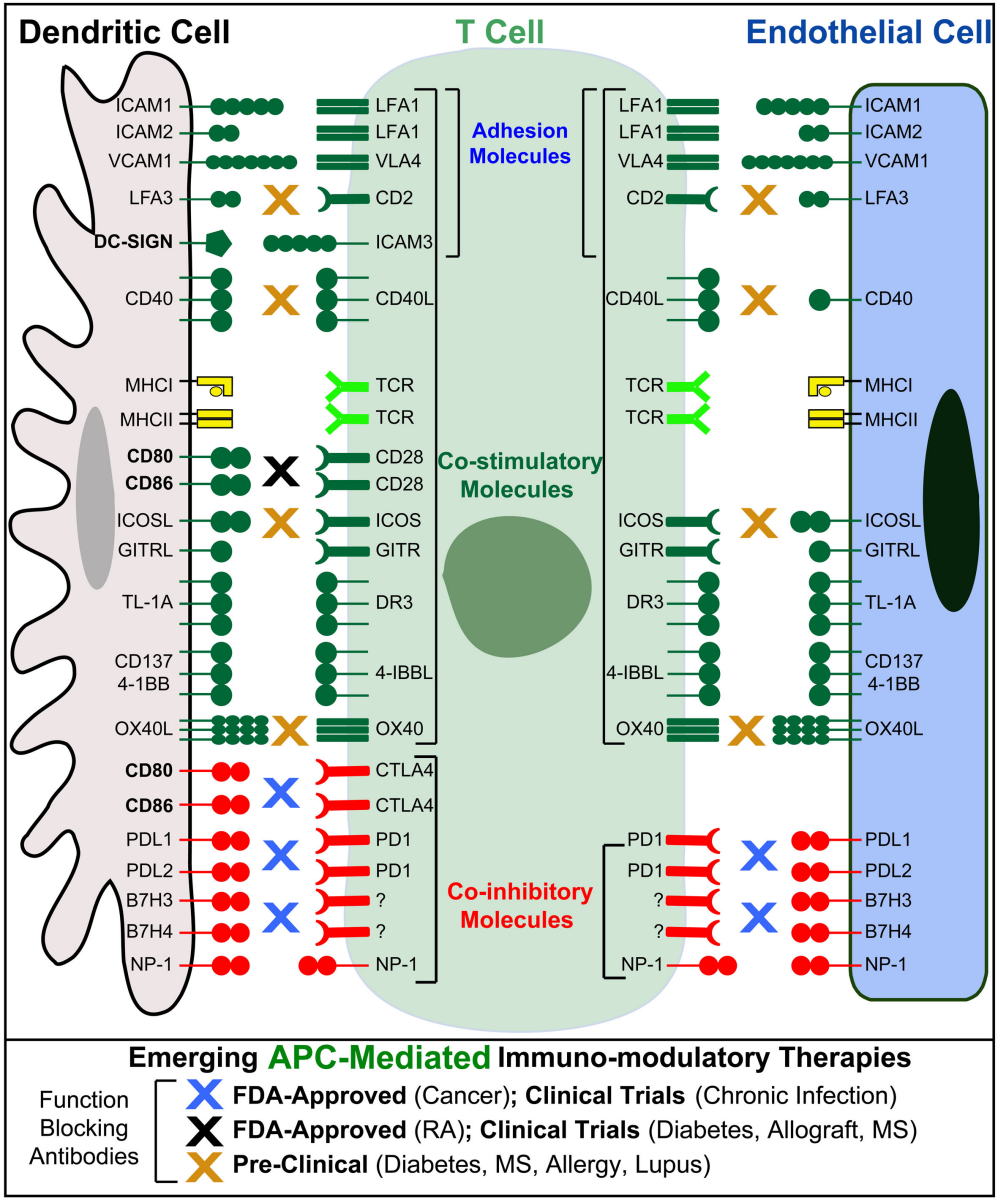
**Endothelial cells as “semiprofessional” non-hematopoietic APCs**. Schematic comparison of the Ag presentation, costimulatory, coinhibitory, and adhesion molecules expressed by dendritic cells (gray) and endothelial cells (blue). Note that endothelial cells, unlike most other non-hematopoietic cells, express most of the critical molecules found in DC express. Important exceptions include CD80 and CD86 that are critical for the activation of naive T cells, as well as the costimulatory/adhesion molecules DC-SIGN. Blue, black, and orange “X”s indicate the possible sites of action for several emerging T cell/APC-directed immunomodulatory therapeutics.

Endothelial cells can express MHC-I and -II both constitutively (though with respect to the latter, some potentially important distinctions may exist between human and mouse, as discussed below) and at higher levels in response to inflammatory cytokines (95, 96) (Figure 3). Endothelial cells also express Ag processing machinery (e.g., LMP2, 7, TAP1, 2, invariant chain, and HLA-DM) and have been shown to efficiently take up, process, and present/crosspresent Ag *in vitro* and *in vivo* (97–103). Moreover, endothelia express a significant range of costimulatory (e.g., ICAM-1, VCAM-1, CD40, LFA-3, ICOSL, 4-1BB, OX40L, and TL1A) and coinhibitory (e.g., PD-L1 and PD-L2) molecules, as well as cytokines, both of which are regulated by inflammatory cues (45, 89, 95, 104–106) (Figure 3). Absent from most endothelia are CD80 or CD86 costimulators that are indispensable for the activation of naive lymphocytes (Figure 3). Thus, endothelial cannot prime naive lymphocytes, but they can readily mediate Ag-specific stimulation of Ag-experienced (i.e., effector/memory) CD4 and CD8 lymphocytes and are therefore regarded as “semiprofessional” APCs (107–112).

The above suggests that the endothelium may provide a unifying nhAPC compartment densely distributed throughout the body and strategically positioned at the blood–tissue interface. At the same time, based on the ability of the endothelium to integrate cues from its microenvironment (9, 10), endothelial APC function should be expected to be locally tuned to uniquely and differentially influence Ag-specific immunity. Indeed, diverse endothelia show heterogeneous expression patterns of MHC-I, MHC-II, coinhibitory and costimulatory molecules (APC surface molecules), and functions in adaptive immune responses. Such local specialization may be roughly analogous to the differentiation of classic hematopoietic APC, such as macrophages into microglia in the brain and into Kupffer cells in the liver.

Before we further discuss the existing observations, however, it is necessary to acknowledge some critical limitations. First, unlike hematopoietic APCs that have been subjected to extensive systematic characterization, the existing knowledge of tissue- and context-specific patterns of endothelial APC surface molecules derives from disparate and often anecdotal observations. In human systems, *in vivo*/*in situ* investigations are limited to a few biopsy- and autopsy-based studies and have usually not included comprehensive panels of the relevant molecules (113–116). Likewise, though it is practically more feasible to conduct the requisite systematic analysis of endothelia in murine tissues, studies and data are still limited. Additionally, while some relatively more detailed characterizations have been conducted *ex vivo* with isolated (largely human and mouse) endothelia, these may not faithfully reflect the tissue-specific features found *in vivo*.

Moreover, it should be noted that some discrepancies in APC surface molecules have been noted in human and mouse, though in light of the above, these require further clarification. *In vitro* both endothelia express MHC-II and both require stimulation by INF-γ in order to do so (95). *In vivo*, however, whereas the microvasculature of most human tissues expresses significant levels of MHC-II constitutively (95, 117), early studies (that focused on coronary artery and microvasculature of the bladder and ureter) (118, 119) questioned whether similar constitutive expression took place in mice. Yet, constitutive expression of MHC-II was subsequently demonstrated in murine liver sinusoidal (45, 120, 121) and lung endothelium (93) and on lymphatic endothelia of LN (122, 123). Moreover, we have recently quantified variable levels of constitutive MHC-II expression in mouse heart, lung, kidney, liver, and skin with the most striking levels found on lung endothelium (Sage and Carman, unpublished observations). Additionally, important differences exist between mice and humans in their endothelial expression of key adhesion and costimulatory and molecules. For example, P-selectin is constitutively expressed by human ECs, where it is sequestered in Weibel–Palade bodies in non-activated endothelium and expressed on endothelia surface following histamine or thrombin presence. Its surface expression is not further upregulated by inflammatory cytokines, as it the case in mice. The costimulatory molecules CD40 and the ICOS ligand GL50 are found on endothelia of humans but not mice (124). While CD58 (a ligand for lymphocyte CD2) is a major costimulatory molecule expressed in human ECs, the analogous ligand in mice CD48 exhibits both dramatically different affinity for CD2 (~50-fold lower) and endothelial distribution (125, 126). Taken together, such factors suggest potentially important species-dependent differences in endothelial APC functions and cautions must be taken in inferring applicability of experimental models to human systems [for review, see in Ref. (124)].

## The T Cell–Endothelial Immunological Synapse

It is has been established that the basic subcellular dynamics at the T cell–APC interface are critical determinants of the responses (127). Studies using either professional APCs or artificial APC models have shown that Ag recognition promotes rapid calcium flux that translates to a migratory arrest and sustained interaction with the APC [i.e., an immunological synapse (IS)]. Microscopic approaches reveal that this is associated with micron-scale clusters of TCR that mediate active signaling (128), which are formed by cytoskeletal modulators, such as Cdc42, WASp, WAVE2, Vav1, Arp2/3, and HS1 (129, 130).

**Figure 4:**
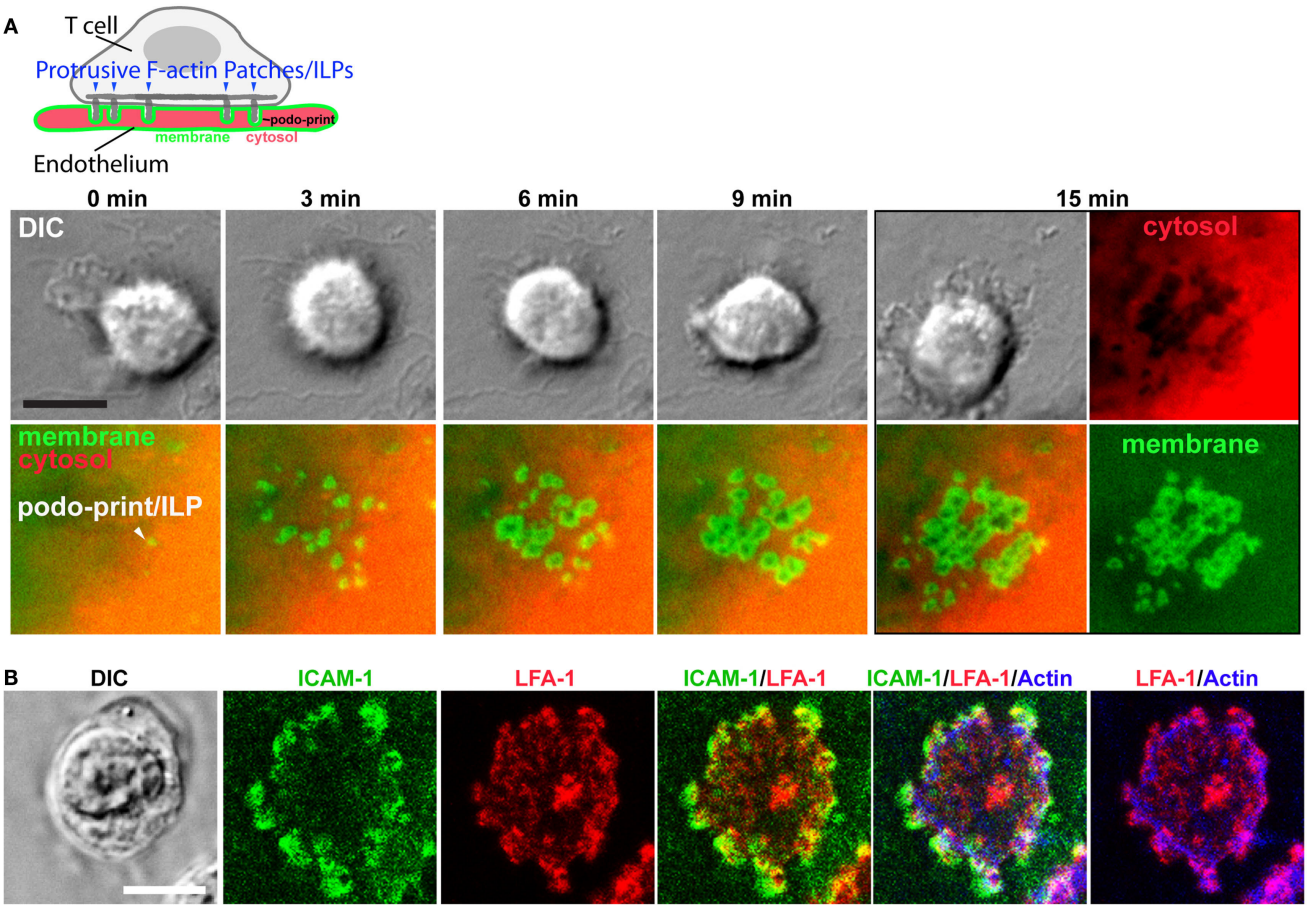
**Imaging the T cell–endothelial immunological synapse (podo-synapse)**. **(A)** Schematic (upper panel) represents formation of stabilized arrays of lymphocyte ILPs protruding into the endothelial surface (note that the labeling of the plasma membrane in green and the cytosol in red corresponds the live-cell imaging experiment, below) following antigen recognition. Images show light microscopy of T cells [by differential interference contrast (DIC) interacting with mem-YFP (green) and cytosol DsRed (ref) transfected ECs]. Podo-prints on endothelium are evidenced as rings of plasma membrane (mem-YFP) where cytosol is excluded (black areas in the “cytosol” image at 15 min). This imaging approach readily reveals the dynamics and discrete three-dimensional architecture of individual ILPs as well as the collective “podo-synapse” ILP array during Ag recognition (112). **(B)** Lymphocytes were incubated with activated, Ag-pulsed endothelium for 5 min, fixed, and stained as indicated and imaged by confocal microscopy. ILPs are enriched in ICAM-1 (green), LFA-1 (red), and Actin (blue), as well as many other immunological signaling molecules (e.g., TCR, MHC-II, PKC-Φ, phyosphotyrosine, and HS1 not shown) (112, 151).

**Figure 5:**
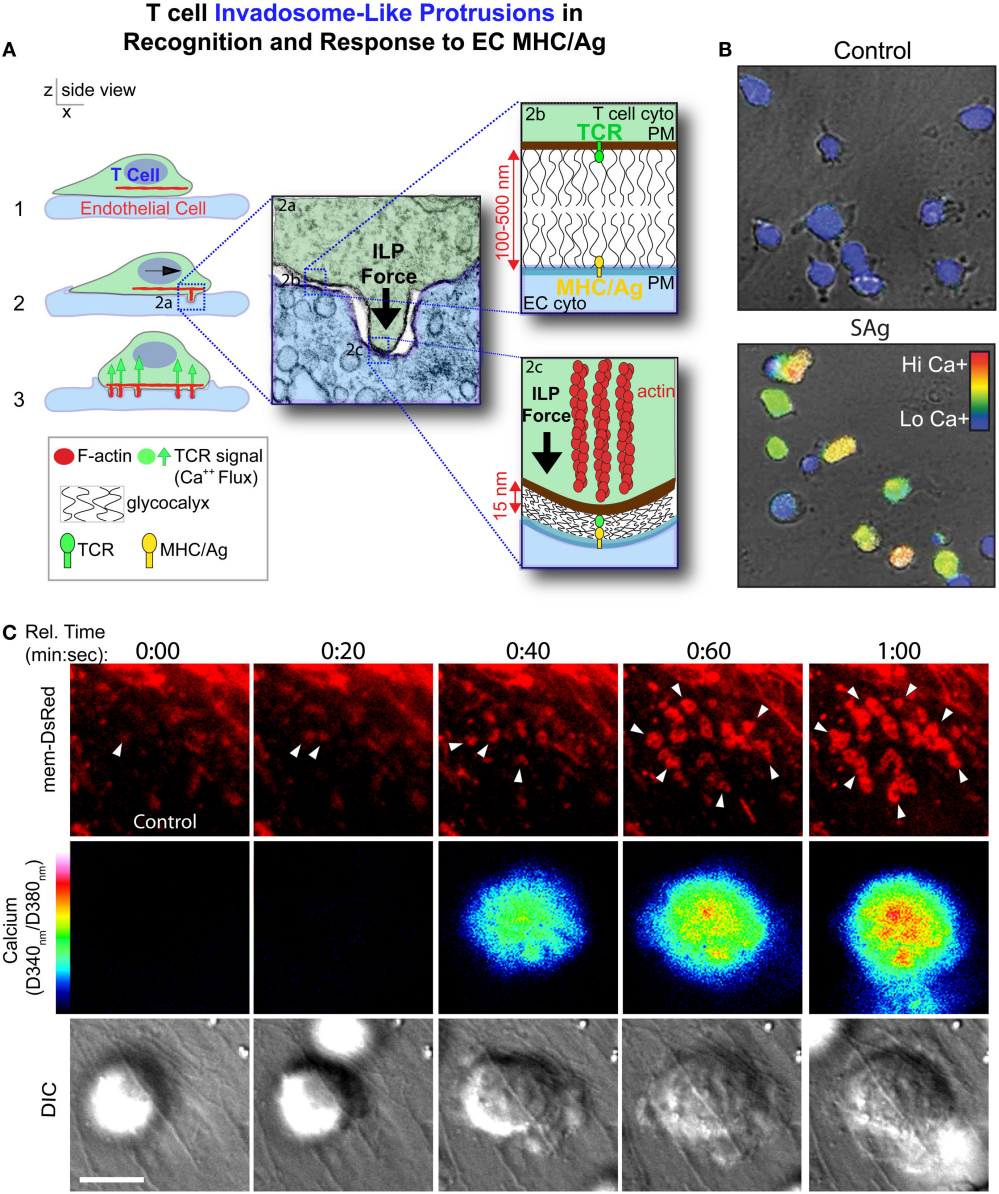
**Model for ILPs function in Ag recognition and response to Ag presented by the endothelium**. **(A)** Schematic shows side views of a memory/effector T cell (green) interacting with the endothelium (blue) presenting cognate Ag. During lateral migration (step 1), lymphocytes dynamically drive ILPs against the opposing cell (step 2, inset 2a). Close interactions between T and APC/target cells, which are partially opposed by the cell glycocalyces (inset 2b), form at ILP tips, facilitating TCR/MHC interactions (inset 2c) in these zones that allows initiation of a response (step 3, calcium flux). **(B)** Avid calcium signaling (Fura-2, rainbow range indicator) response for effector/memory T cells migrating on endothelial presenting cognate antigen (lower panel) but not antigen-negative control endothelial (upper panel) (112). **(C)** T cells were labeled with Fura-2 and imaged live migrating on Ag-pulsed, mem-DsRed transfected endothelium. Upper panels show mem-DsRed. Arrows indicate initial ILP formation (see rings of fluorescence; see also Figure 4A). Middle panels indicate calcium flux values on a rainbow scale. Lower panels show the DIC image of the T cell–EC interaction. Note that as in **(A)**, initial ILPs (read out hear by visualization of the cognate podo-prints) preceded the initiation of calcium flux which follows shortly after and accumulation of stabilized ILPs (i.e., formation of a podo-synapse) occurs commensurate with peak calcium flux (112).

In the first detailed dynamic imaging investigation of the T cell–endothelial ISs showed that in diverse *in vitro* models (i.e., using both human and mouse freshly isolated CD4 Th1 effector memory, IL-2-activated CD4 Th1, and CD8 CTL along with dermal and lung microvascular endothelium and either peptide or super-antigens) endothelial Ag presentation was shown to consistently induce a sustained (30–60 min) calcium flux that was coupled to a transient arrest in migration and nuclear translocation of NFAT (112). Close characterization of the T cell–EC IS revealed that initiation of T cell probing by ILPs (Figure 4) consistently preceded, and was required for, Ag recognition and the triggering of calcium flux (Figure 5). This suggests that the “informational scanning” roles of ILPs discussed above [i.e., in relation to specific pools of endothelial surface chemokines (61)] are also relevant for detection of MHC/Ag. As noted above, the glycocalyx [a 50–500 nm thick polysaccharide coating found on all cells (131, 132) that stabilizes the plasma membrane (133)] provides a barrier to close intercellular membrane–membrane encounter. As such, small cell surface adhesion and signaling molecules [e.g., TCR and MHC, each ~7 nm tall (134)] are effectively shielded (131, 132, 135–137) (Figure 5A). In this way, the glycocalyx inhibits, or at least limits, immune cell adhesion and immune recognition (138–147). Protrusive forces provided by ILPs overcome this barrier, driving close membrane–membrane apposition (Figure 5A) and thereby promoting molecular interactions that might otherwise be inefficient or impossible. It is interesting to note that while the leading edge lamellipodia of T cells have been well known to possess heightened Ag recognition sensitivity (148–150), this very same subcellular region is now shown to be the preferential site of ILP formation (56, 60, 112). Thus, ILPs could be regarded as specialized “actuators” of immune surveillance.

Following initial Ag recognition, the resulting intracellular calcium flux induces accumulation of stabilized clusters of ILP that generate a unique IS topology termed a “podo-synapse” (112) (Figures 4 and 5). Here individual Ag-stabilized ILPs become enriched in actin, TCR, and other molecules suggestive of active local signaling (e.g., PKC-Φ, phospho-tyrosine, and HS1) (Figures 4 and 5). Thus, ILPs may promote sustained TCR signaling by providing sub-micron-scale volumes that have “signalosome” qualities for increased signaling efficiency (152–156) analogously to the signaling microclusters and “multifocal” ISs that have been defined in planar lipid bilayer APC model (157–164) and on DCs (157–159), respectively. Additionally, the collective architecture formed by the arrays of stabilized ILP arrays that form may serve other functions. Specifically, the podo-synapse exhibits striking topological similarity to osteoclast “podosome-belts” (165, 166) (Figures 4 and 5), structures that form sealing zones for directed secretion of bone-degrading enzymes (167). Thus, the podo-synapse architecture could be envisioned to function analogously, that is, to direct concentrated secretion of cytokines or cytotoxic agents toward the target cell, while limiting bystander effects.

As discussed in the following section, one of the characterized functional roles for endothelial Ag presentation is Ag-specific recruitment of T cells (168–173). Studies examining freshly isolated human CD4 effector memory and human dermal microvascular endothelial cells presenting super-antigen, further defined distinctive structures involved in such TCR-regulated trafficking (168). Specifically, large “transendothelial protrusions” (TEPs) were found to develop in an Ag-, TCR-, ICAM-1-, and fractalkine-dependent and costimulatory molecule-independent manner in T cells positive for nuclear NFAT (168). These TEPs were evident extending through the endothelial junctions and therefore are thought to be the first step in Ag-specific transendothelial migration. Following completion of diapedesis, lymphocytes (as well as all other leukocytes) spend a significant amount of time (5–20 min) in the abluminal space (i.e., between the endothelium and the underlying basement membrane) before they enter the tissue and begin their search form interstitial APCs or targets. There are many potential consequences of these T cell/EC interactions (explored further in later sections): for instance, we know that EC Ag presentation can induce lasting effects on T cells (e.g., nuclear NFAT translocation, altered surface expression of activation markers, cytokine expression, and proliferation). This would suggest that responses to immediately subsequent interactions with interstitial APCs and targets might be altered (e.g., primed, suppressed, and phenotypically skewed). Yet, such consequences have to be directly determined. Likewise, it seems reasonable to expect that similarly to other APCs, endothelia receive “help” through their Ag-specific ISs with CD4 lymphocytes. The ability/importance, for example, of CD40 in providing signals to APCs is clearly illustrated by B cells, which depend on lymphocyte CD40L engagement to stimulate proper immunoglobulin responses (174). CD40 is also expressed on endothelial cells (in humans) (104, 175) and stimulation of endothelial CD40 promotes strong endothelial activation, as demonstrated by upregulated expression of E-selectin, ICAM-1, and VCAM-1 and increased leukocyte adhesion (104, 176, 177). Thus, one might hypothesize that sustained CD40 ligation resulting from Ag recognition might induce endothelial activation that influences subsequent recruitment of other (e.g., innate) immune effectors.

## Potential Functions of Endothelial nhAPC

While overall roles for endothelial cells in adaptive immunity remain to be completely elucidated, it is clear that their functions will be ancillary to those of professional APCs (95, 178). Nonetheless, as discussed above, unique non-redundant functions for nhAPC have been established. Endothelia are perhaps the most abundant APC in the body [outnumbering the professional APC compartment by ~1000-fold (39, 179)], and they are strategically positioned to make constant contacts with circulating lymphocytes. As such, endothelia would seem to have excellent opportunity to exert qualitatively distinct and locally tuned peripheral regulation of adaptive immune responses. As summarized below, such regulation may be highly varied reflecting the highly heterogeneous contexts in which they develop.

Given the central function of endothelium in regulating trafficking of immune cells, it may not be surprising that multiple studies have shown that Ag presentation can alter trafficking of T cells bearing the cognate TCR, whereas some studies evidence Ag-mediated migratory stop signals that prevent or delay diapedesis (112, 180, 181), others support the intriguing hypothesis that Ag presented on the endothelium promotes the selective diapedesis (and therefore recruitment) of Ag-reactive T cells (168–173). Along these lines, a recent study demonstrate that recruitment of Treg to sites of inflammation is strongly favored by their ability to recognize self-Ags presented by endothelium (182). Of note, the specific expression patterns of costimulatory/coinhibitory molecules found on endothelium are thought to strongly influence the above processes (183).

Other studies have evidenced ability of endothelial antigen presentation to either drive inflammation or promote tolerance. Regarding the former, resting CD4 memory cells have been shown *in vitro* and *in vivo* to become activated to proliferate and secrete inflammatory cytokine (e.g., IFN-γ, IL-2, IL-4, and IL-10) in response to Ag (or allo-Ag) presented by endothelium in an MHC-II- and costimulator-dependent manner (107–109, 112, 184–186). Moreover, presence of MHC-I-bearing cognate Ag on endothelia promotes CD8 CTL activation and endothelial cell killing, in a coinhibitor (i.e., PD-L1 and PD-L2)-modulatable fashion (89, 105, 111, 112). Additionally, through their ability to capture and retain Ag (a process called “archiving”) LEC they have been shown to help maintain protective CTL memory (187).

The idea that ECs can provide a form of peripheral tolerance (e.g., “transmigration anergy”) has been evidenced in diverse settings (188, 189). In the liver, a well-known strongly tolerogenic environment, the liver sinusoidal ECs (LSECs) exhibit extremely efficient scavenging, crosspresentation (100, 101, 190), and unique tolerogenic functions (191, 192). For example, LSEC promotes Ag- and coinhibitor-dependent CTL tolerance (102), suppression of CD4 proinflammatory Th1 and Th17 responses (31, 193, 194), and differentiation of CD4 Tregs (195). Additional endothelia with particularly prominent tolerogenic properties are those that line the lymphatic vessels and the contiguous sub-capillary, cortical, and medullary sinuses of the SLOs (LECs) (45, 103, 121). Through absent costimulatory and high coinhibitory (i.e., PD-L1) molecule expression, crosspresentation of Ag to CTLs causes clonal deletion of self-reactive lymphocytes (196, 197). As T cells must cross the cortical and/or medullary LECs in order to egress from the LN, these LECs are proposed to serve as anatomical checkpoint to enforce self-tolerance in lymphocytes as they attempt to egress from the LN (121).

## Roles for Endothelial nhAPC in Disease

Most diseases have a significant inflammatory component and as such are inherently linked to alterations in endothelial phenotype and function along with and altered trafficking of immune cells. Significant circumstantial evidence suggests the endothelial nhAPC functions are also altered in disease. Notably, endothelial expression of MHC-II is greatly upregulated in multiple autoimmune/inflammatory diseases including allograft rejection, diabetes, dilated cardiomyopathy, myocarditis, multiple sclerosis, rheumatoid arthritis, lupus, vasculitis, and Crohn’s disease (113, 114). Moreover, changes in endothelial expression of key costimulatory and coinhibitory molecules (e.g., CD40 and PD-L1) have been linked with pathogenesis in multiple inflammatory and autoimmune diseases, as discussed below (89, 104–106). It remains to be determined, however, whether these changes are functionally relevant and, if so, whether they are contributing to pathogenesis or are rather acting to limit it. Below, we summarize several settings whereby endothelial nhAPC functions seem relatively well established to be promoting pathogenesis.

### Allograft Rejection

Functional roles of EC MHC are perhaps best evidenced in allo-response, which have clearly been demonstrated in the extensive *in vivo* models and clinical investigation of transplantation (163, 198–200). *In vitro* CD4 and CD8 effector/memory T cells are also well evidenced to be directly activated by allogeneic endothelium (163, 164). Likewise, *in vivo* the endothelium of solid organ transplants (which are the first cells that host lymphocytes encounter) plays a critical MHC-dependent role in promoting allo-responses and also can become a major target for CD8-mediated injury. Additionally, in studies of graft-versus-host disease (i.e., in setting of stem cell transplantation), allo-reactive donor CD8 CTLs were shown to be directly responsible for endothelial damage (201).

### Diabetes

Studies have shown *in vitro* that human endothelial cells can take up and process the type I diabetes islet autoantigen GAD65 and functionally present it on MHC-II molecules, which induces selective transmigration of Ag-specific Th1 lymphocytes (97). Additionally, the expression level of MHC-I on endothelial tissues has a direct impact upon the efficiency of migration of autoreactive T cells *in vivo* (202). Similar studies in a murine model of type I diabetes demonstrated that insulin-specific CD8 lymphocytes home to the pancreas in a manner that was dependent on endothelial presentation (173). These findings suggest a pathogenic model whereby the trafficking and nhAPC roles of the endothelium conspire to promote local enrichment of autoreactive proinflammatory lymphocytes.

### Multiple Sclerosis

Initial studies in Experimental Autoimmune Encephalitis (an animal model of MS) made the observation that T cell infiltration into the brain was consistently preceded by elevated MHC-II expression on brain microvascular endothelium. This suggested the idea that EC Ag presentation might have a causal role in generating such infiltrates (203). Further work showed that MHC-I/Ag presented on the luminal surface of the blood–brain barrier was functionally responsible for Ag-specific trafficking of T cells to the brain (204). *In vitro* studies have also demonstrated that human brain endothelial cells constitutively coexpress of MHC-I, MHC-II, CD40, and ICOSL, readily take up fluorescently labeled Ags via macropinocytosis and drive Ag-dependent proliferation of CD4 and CTLs (198).

### Cerebral Malaria

Endothelial antigen presentation in the development and progress of cerebral malaria has been implicated both by the observation that deposition of *Plasmodium* antigens could be detected in autopsies of patients that die of the disease and by the fact that *Plasmodium*-derived lactate dehydrogenase or pAldose was detected in the blood vessels of brain, heart, and lung, specifically inside endothelial cells (205). Additionally, *in vitro* studies have shown that brain endothelial cells can take up Ag from *Plasmodium*-infected red blood cells and can activate CD4 and CD8 lymphocytes (205). Recent studies in the murine model of cerebral malaria caused by *P*. *berghei* ANKA (PbA) showed that endothelial cells are the population of nhAPC responsible for crosspresentation PbA antigen *in vivo* (rather than pericytes or microglia) and that PbA antigen crosspresentation by primary brain endothelial cells *in vitro* confers susceptibility to killing by CD8^+^ T cells from infected mice (199).

## Endothelial Cells as Putative Mediators of APC-Targeted Immunomodulation

Antigen-presenting cell-targeted immunomodulatory therapy is an extremely exciting and promising frontier (200, 201, 205–211). Therapies targeting costimulators and coinhibitors that can have either adjuvant or tolerizing function have become an enormously important new strategy. For example, blocking antibodies that disrupt the PD-1/PD-L coinhibitory axis represents an innovative new “tolerance-breaking” treatment for cancer and excess/chronic infection (200, 201, 205–207).

However, significant incidence of severe inflammatory pathology still exists with such approaches (208–212). It is recognized that a better understanding of precisely where/how (i.e., on which APCs) these drugs act is necessary to improve their utility for these and many other diseases (208–212). A range of studies have demonstrated that critical and distinct effects of PD-1/PD-L1 blockade are contributed through peripheral nhAPCs, and several of these investigations strongly imply endothelial cell specifically in such effects (82, 83, 85, 86, 89, 92, 207, 213).

Additionally, a series of clinical and *in vitro* studies suggest that therapeutic effects of statins on atherosclerosis (214–219) and rapamycin on cardiac transplant rejection (220) may be through altering endothelial expression of PD-L1 and CD40. However, putative roles for endothelia APC in mediating effects of these immunomodulatory drugs have yet to be directly characterized. The fact that endothelial are perhaps the most abundant [i.e., outnumbers the professional APC compartment by ~1000-fold (39, 179)] and bioavailable APCs in the body would suggest that a better understanding of endothelia as APC and as putative targets for immunomodulatory therapy is warranted (201).

## Summary

While the endothelium provides an as essential barrier between the blood–lymph circulation and the tissues, it also functions as an active regulator of immune function. Specifically, through expression and presentation of chemoattractants and adhesion molecules and cytoskeletal remodeling, the endothelium plays fundamental role in directing the selective trafficking of immune cells in and out of tissues. Additionally, through expression of MHC-I, MHC-II, and a wide array of costimulatory molecules, endothelial exhibit “semiprofessional” APC functions that can communicate Ag-specific information to effector/memory T cells in the periphery. Furthermore, evidence from diverse models presents an emerging picture of endothelia as locally tuned functionally heterogeneous APCs that instruct highly context dependent responses. While these functions are ancillary to those of professional APCs, the large scale and strategic anatomical positioning of the endothelial nhAPC compartment suggests important, non-redundant functions in peripheral in adaptive immune responses that should be considered in the context of emerging APC-directed immune-modulatory therapeutics.

## Author Contributions

CVC and RM cowrote the manuscript and prepared the figures.

## Conflict of Interest Statement

The authors declare that the research was conducted in the absence of any commercial or financial relationships that could be construed as a potential conflict of interest.
